# Hispaniolan Hemilophini (Coleoptera, Cerambycidae, Lamiinae)

**DOI:** 10.3897/zookeys.258.4391

**Published:** 2013-01-15

**Authors:** Steven W. Lingafelter

**Affiliations:** 1 Systematic Entomology Laboratory, Agriculture Research Service, United States Department of Agriculture, National Museum of Natural History, Washington, D.C. 20013-7012, U.S.A.

**Keywords:** Longhorned woodborers, Hispaniola, Haiti, Dominican Republic, endemic, Batesian mimicry

## Abstract

The Tribe Hemilophini (Lamiinae) is reviewed for Hispaniola and an identification key is provided. Fifteen species are now known from the island, including one new species of *Adesmus* (*Adesmus fortunei* from Pedernales and La Vega Provinces, Dominican Republic), one new species of *Oedudes* (*Oedudes anulatus* from Peravia and La Vega Provinces, Dominican Republic), and five new species of *Calocosmus* (*Calocosmus contortus* from San Cristóbal Province, *Calocosmus punctatus* from Peravia Province, *Calocosmus rawlinsi* from Elías Piña Province, *Calocosmus robustus* from La Vega Province, and *Calocosmus thonalmus* from La Altagracia Province, all in the Dominican Republic). *Oedudes* and *Adesmus* are new island and country records for Hispaniola and Dominican Republic, respectively. *Calocosmus holosericeus* Gahan is a new synonym of *Calocosmus janus* Bates. In addition to the new species, five new country records and four new island records are presented for *Calocosmus*.

## Introduction

**Figure 1.**
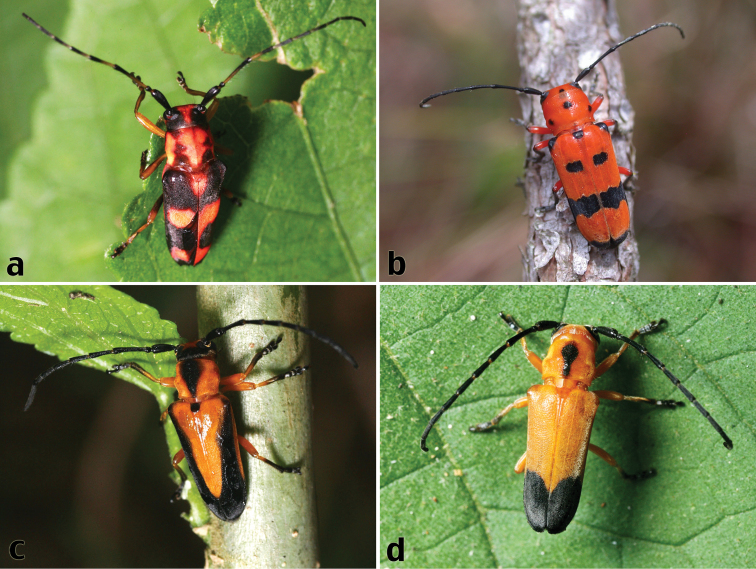
Live habitus photos of Hispaniolan Hemilophini: **a** *Oedudes anulatus* Lingafelter, sp. n. (La Vega Province) (photo by Rick Stanley) **b** *Calocosmus magnificus* Fisher (Pedernales Province) (photo by Kelvin Guerrero) **c** *Calocosmus nigritarsis*, unusual morphotype (La Vega Province) (photo by Rick Stanley) **d** *Calocosmus nigritarsis*, typical morphotype (Independencia Province) (photo by Gino Nearns).

**Figure 2.**
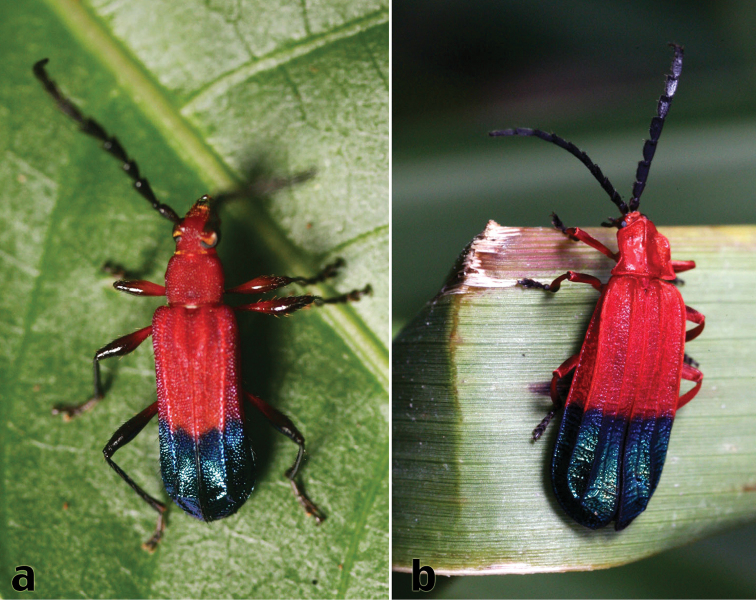
Members of the Batesian mimicry complex with Hispaniolan Hemilophini: **a** *Trichrous terminalis* (White) **b** *Thonalmus* sp. (Lycidae). Photos by Rick Stanley.

The Hemilophini of Hispaniola are recognized by their deeply bifid tarsal claws, very broadly expanded prosternal intercoxal process, very deeply notched eyes, vestiture of very dense, short, often scale-like pubescence over much of the body, and typically bright, aposematic colors (Fig. 1a–d). Many of the species, along with *Trichrous* species (Cerambycinae: Heteropsini) (Fig. 2a), are presumably Batesian mimics of *Thonalmus* Bourgeois (Fig. 2b), a genus of Lycidae that is common on Hispaniola and is noxious to lizards (Darlington 1938). The Hemilophini are diverse in the Neotropics with species most abundant in Central and South America. The tribe is represented by three extant genera (*Adesmus* Lepeletier, *Oedudes* Thomson, and *Calocosmus* Chevrolat) and one extinct genus (*Paleohemilophus* Martins & Galileo) on the island of Hispaniola (Monné and Bezark 2011). *Calocosmus*, a genus endemic to the West Indies (Peck and Perez-Gelabert 2012), has radiated in Hispaniola, with 13 of the 18 species (72%) (including 5 new ones) occurring there (Perez-Gelabert 2008; Monné and Bezark 2011). *Adesmus* and *Oedudes* are recorded for Hispaniola for the first time with one new species each, described herein.

## Methods

Specimens from the following institutional and private collections (with acronyms used in this paper) were examined for this study:

ACMT American Coleoptera Museum, San Antonio, TX, U.S.A. (J. Wappes)

BMNH The Natural History Museum, London, England (M. Barclay)

CMNH Carnegie Museum of Natural History, Pittsburgh, PA, U.S.A. (J. Rawlins, R. Davidson, R. Androw)

DRMC Museo Nacional de Historia Natural, Santo Domingo, Dominican Republic (G. de los Santos)

EFGC Edmund F. Giesbert Collection, Gainesville, FL, U.S.A. (at FSCA, M. Thomas)

ENPC Eugenio Nearns Private Collection, Albuquerque, NM, U.S.A.

FSCA Florida State Collection of Arthropods, Gainesville, FL, U.S.A. (M. Thomas)

CRAAG Centre de Recherches Agronomiques Antilles-Guyane, Duclos (Petit-Bourg), Guadeloupe (ex-collections Fortuné Chalumeau)

MNHN Museum National d’Histoire Naturelle, Paris, France (G. Tavakilian)

RHTC Robert H. Turnbow, Jr. Private Collection, Ft. Rucker, AL, U.S.A.

TAMU Texas A&M University Collection, College Station, TX, U.S.A. (E. Riley)

USNM National Museum of Natural History, Smithsonian Institution, Washington, DC, U.S.A. (S. Lingafelter)

WIBF West Indian Beetle Fauna Project, Bozeman, MT, U.S.A. (M. Ivie)

Photographs of primary types from the following institutions were also examined:

AMNH American Museum of Natural History, New York, NY, U.S.A. (Lee Herman)

BMNH The Natural History Museum (London, United Kingdom) (M. Barclay)

MCZC Museum of Comparative Zoology, Harvard University, Cambridge, MA, U.S.A. (B. Farrell, P. Perkins)

MNHN Museum National d’Histoire Naturelle, Paris, France (G. Tavakilian)

Holotypes of the new species are deposited in USNM and CMNH. Label data is semi-verbatim with line spaces separated by commas and abbreviated localities and dates spelled out for clarity. Measurements were made using Axiovision software (version 4.8.2) on images taken with a Zeiss AxioCam HRc camera attached to a Zeiss Discovery.V20 stereomicroscope with Sycop motorized zoom and focus control and a PlanApo S 0.63× objective. Diagnoses, figures, distributional and phenological records are presented for all Hispaniolan Hemilophini, along with full descriptions for the new species.

## Key to Hemilophini of Hispaniola

(does not include the extinct *Paleohemilophus dominicanus* Martins & Galileo)

**Table:** 

1	Third antennomere and base of fourth yellow, remainder dark purple to black; pronotum without any visible punctation, covered in dense vestiture of pale greenish-white pubescence	*Adesmus fortunei* Lingafelter, sp. n.
–	Antennomeres differently colored; pronotum with at least a few obvious punctures	2
2(1)	Elytral apex bidentate or subspinose, with an acute projection at suture and outer angle	*Oedudes anulatus* Lingafelter, sp. n.
–	Elytral apex broadly or narrowly rounded, lacking any spines or acute projections (*Calocosmus*)	3
3(2)	Elytron mostly uniformly black or purplish-black; fulvous or orange regions (if present) along suture, base, and/or lateral margin	4
–	Elytron not mostly uniformly black or with purplish tinge; bicolored with dark black or blue and orange or red maculae; fulvous regions in different pattern than above	7
4(3)	Humerus prominent (elytron noticeably wider at base than apex)	5
–	Humerus not prominent (elytron not noticeably wider at base than apex)	6
5(4)	Antenna completely black, without basal annulations of gray pubescence; elytron completely dark purplish-black, with moderately well defined or poorly defined punctures	*Calocosmus janus* Bates
–	Antenna with basal annulations of gray pubescence on most antennomeres; elytron black except at base, along anterior part of suture, a small spot at middle, and part of lateral margin which are fulvous; elytral punctures dense and well-defined except at extreme apex	*Calocosmus punctatus* Lingafelter, sp. n.
6(4)	Elytral suture not pale colored; pronotum transversely bisulcate (not always obvious)	*Calocosmus nigripennis* Chevrolat
–	Elytral suture pale colored; pronotum not transversely bisulcate	*Calocosmus semimarginatus* Bates
7(3)	Apex of elytron strongly modified with two elevated, longitudinal ridges lined with bristly pubescence	8
–	Apex of elytron without elevated, pubescent, longitudinal ridges.	9
8(7)	Antennae uniformly black, without annulations; scutellum without black setae; eye of male small; lower eye lobe removed from genal margin by over two-thirds of its height	*Calocosmus thonalmus* Lingafelter, sp. n.
–	Antennae with antennomeres 4–7 orange annulate at the base; scutellum with numerous, short, black setae; eye of male large; lower eye lobe removed from genal margin by less than one-third of its height	*Calocosmus contortus* Lingafelter, sp. n.
9(7)	Apical macula of elytron restricted to tip, very small, less than apical one-eighth; pronotum dorsally with three small, dark maculae (one at center, and one at each side of center), also a small dark macula laterally on each side; humerus with small, isolated macula	*Calocosmus magnificus* Fisher
–	Apical fourth to half of elytron with large, black or purplish macula; pronotum either without dark macula, or macula restricted to middle; humerus without isolated macula (if dark, part of a larger maculate region extending over more of elytral base than just humerus)	10
10(9)	Apex of elytron with dark macula mostly shiny, iridescent, and glabrous	*Calocosmus hispaniolae* Fisher
–	Apex of elytron with dark macula mostly pubescent, not shiny, with or without an iridescent luster	11
11(10)	Humerus not prominent (not projecting anterolaterally; usually without glabrous region; elytron approximately parallel-sided); pronotum with weak lateral and dorsal swelling at middle; relatively delicate species (specimens < 4 mm wide); pronounced hair patches present on posteromedial part of metasternum in males	12
–	Humerus prominent (projecting anterolaterally; usually with small glabrous region at apex; elytron often noticeably wider at base than apex); pronotum with pronounced lateral and dorsal swelling at middle; robust species (most specimens, particularly females, > 4 mm broad); conspicuous hair patches absent on posteromedial part of metasternum in males	13
12(11)	Elytral base, including scutellum, nearly completely dark; legs nearly completely dark reddish-brown to black; apical black macula of elytron without punctures except at extreme anterior margin	*Calocosmus rawlinsi* Lingafelter, sp. n.
–	Elytral base fulvous, or if black macula present at base, it does not extend to suture or scutellum; scutellum fulvous; femora fulvous, tibiae and tarsi variable, either fulvous or dark reddish-brown; apical black macula of elytron with punctures present except at extreme posterior margin	*Calocosmus chevrolati* Fisher
13(11)	Tibiae fulvous; tarsi fulvous or slightly darkened; venter fulvous; base of elytron never with dark macula; antennomeres (4–7 at least) with fulvous basal annulation, otherwise black; dorsum of pronotum and elytron with a short, dense vestiture of pale, ashy pubescence visible from some angles but inconspicuous from other angles (more pronounced on fresh specimens)	*Calocosmus melanurus* Gahan
–	Tibiae and tarsi partially or completely dark brown to black; venter variably colored, but rarely all fulvous; metasternum typically with a black macula, sometimes most of venter brown to black; base of elytron with or without dark macula; antennae variably colored, either all black or with basal fulvous annulations; distinctive ashy pubescence of elytron and pronotum absent	14
14(13)	Antenna all black (occasionally antennomeres 4–6 with narrow basal fulvous annulation); pronotum with large, dark macula in center, broader anteriorly, in most specimens (occasionally reduced to small anteromedial spot)	*Calocosmus nigritarsis* Fisher
–	Antenna with most antennomeres (usually 3–9, at least) with basal fulvous annulation; pronotum entirely fulvous or with very small anteromedial dark macula	*Calocosmus robustus* Lingafelter, sp. n.

## Taxonomy

### Adesmus fortunei

Lingafelter sp. n.

urn:lsid:zoobank.org:act:6ABC8A11-F1C8-4F4B-BA67-89FA7BDABE4E

http://species-id.net/wiki/Adesmus_fortunei

Fig. 3a ; Map 1

#### Diagnosis.

This species is unlike any other species in the Caribbean Islands. It is very similar to *Adesmus nigrocinctus* Gahan, from Brazil, in the overall form, proportions, and antennal and elytral pubescence. It is distinguished from all other Hispaniolan hemilophines by the very long third antennomere, which, along with the basal half of the fourth is yellow, the densely pubescent pronotum without evident punctures, and the unique pattern of alternating white and black transverse fasciae on the elytra.

#### Description.

Size: 9.7–13.8 mm long; 3.5–5.1 mm wide between humeri. *Head* with dense vestiture of appressed off-white setae, thickened at base, almost scale-like; most dense on frons, less dense around eyes, base of antennal tubercles and vertex. Punctures indistinct or hidden by pubescence. Frons not bulging, level to slightly convex between eyes with exception of small medial impression extending to vertex. Gena below lower eye lobe and mandibular base a little more than one-third height of lower eye lobe; frontal-genal ridge incomplete, not extending to eye margin; ante-clypeal sulcus transverse, but mostly hidden by pubescence. Eye large, bulging laterally on lower lobe, finely faceted, upper lobe connected to lower lobe by 3–4 facets at narrowest point, lower lobe much larger than upper lobe, occupying most of head from lateral view. Interantennal region moderately impressed with antennal tubercles moderately elevated. Antenna of female extending beyond elytral apex by 2 antennomeres (by 3–4 antennomeres in males). Antenna with fringe of denser, short pubescence of two colors, white and black, and scattered, less dense, longer setae. Antennomeres black with exception of third and basal one-fourth to one-half of fourth which are yellow. Antennomere 3 very long (longer than scape + 2, nearly as long as 4+5), subsequent antennomeres gradually decreasing in length. *Prothorax* cylindrical, much broader than long (1.8–2.8 mm long; 2.8–3.8 mm wide), distinctly narrower than elytral base, with very slight lateral swelling, densely covered with very pale green (nearly white) scale-like pubescence covering integument and any punctures; pronotum without dorsal calli or tubercles. Pronotum about one-fifth length of body. Prosternum densely pubescent with short, appressed, off-white setae; prosternal process broadly expanded at apex, closing procoxal cavities posteriorly. *Elytron* with distinct punctures on basal third, becoming shallow at middle, and inconspicuous or absent at apical third, bold, transverse purplish-black fasciae at basal fourth, just posterior to middle, and at apex. These transverse areas covered with narrow, suberect, non-scale-like black setae. Between and around the purplish-black fasciae are broad, transverse areas covered with short, dense, scale-like white or off-white setae. Humeri not projecting. Elytral apices narrowly rounded to suture, without spines. Elytron 7.2–9.7 mm long; 1.7–2.7 mm wide; elytral length/width: 3.7–4.2. *Scutellum* narrowly rounded posteriorly, with sparse pale green or white pubescence. *Legs* with femora and tibiae sublinear, only weakly thickened apically. Metafemur short, extending to about third ventrite, with moderately dense white and black pubescence not obscuring surface. Legs bicolored with protibia and venter of profemur yellow, mesotibia and metatibia yellow, and base of tarsomeres 1 and 2 yellow, all remaining portions of legs black. *Venter* mostly densely pubescent with metathorax and last 3 ventrites mostly covered in short, white pubescence, elsewhere mostly covered in black pubescence. Apex of fifth ventrite of males and females broadly truncate with small median notch.

**Figure 3.**
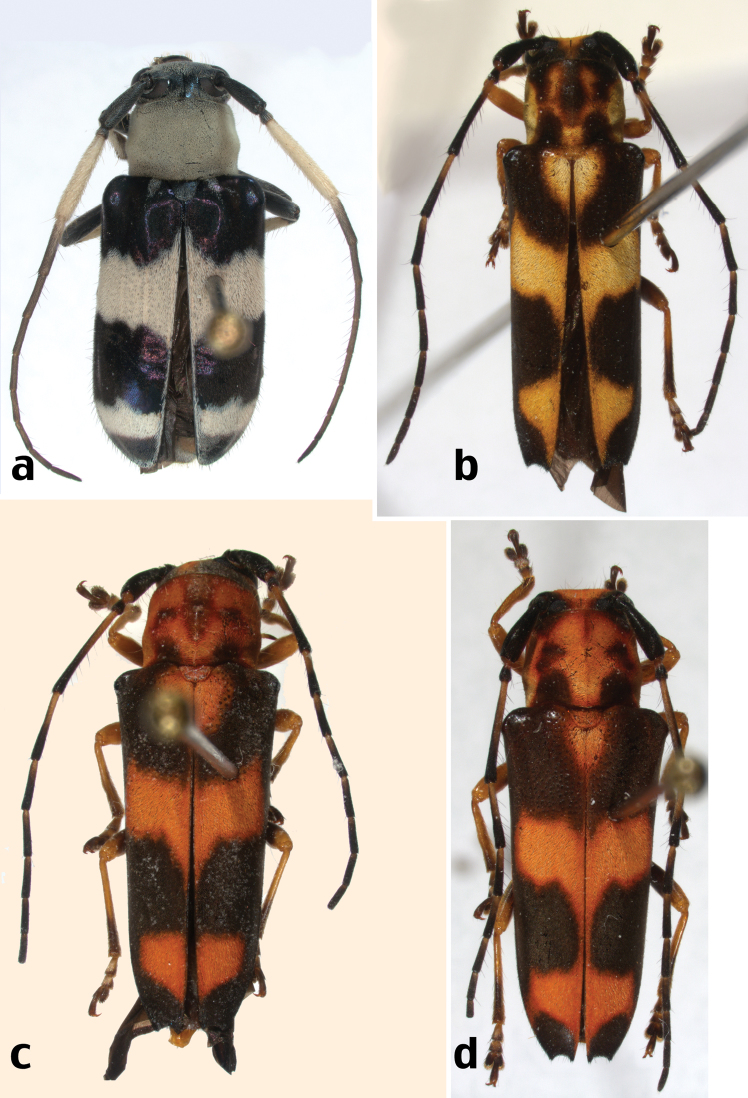
New species of Hispaniolan Hemilophini: **a** *Adesmus fortunei* Lingafelter, sp. n., holotype **b** *Oedudes anulatus* Lingafelter, sp. n., holotype **c** *Oedudes anulatus* Lingafelter, sp. n., paratype **d** *Oedudes anulatus* Lingafelter, sp. n., paratype.

**Map 1.**
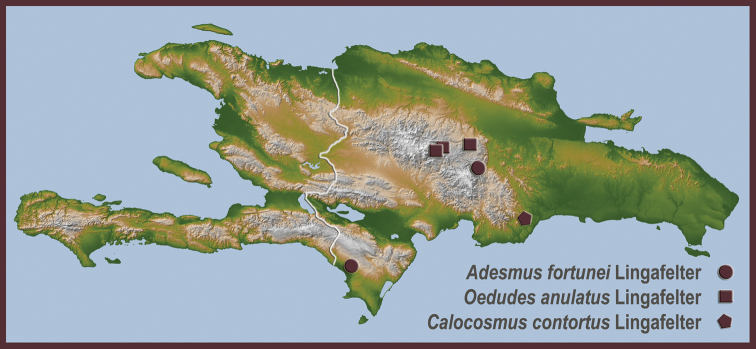
Distributional locality records for new species of Hispaniolan Hemilophini.

#### Etymology.

The species epithet is a genitive patronym in honor of Fortuné Chalumeau (CRAAG) for his extensive and important work in Cerambycidae of the Lesser Antilles and for collecting the first known specimen.

#### Notes.

*Adesmus* is represented by more than 50 species in Central and South America, but this species represents the first occurrence of the genus in Hispaniola and only the third one known from the Caribbean Islands. It is known from two specimens.

#### Material.

Holotype (female): Dominican Republic: Pedernales Province, N. of Cabo Rojo; Parque Nacional Sierra de Bahoruco; km 26 on Carretera Arcoa [sic]; transitional forest (between dry and pine forests); elev. 691 m; 18.113500°, -71.621100°, 11 May 2010, coll. G.J. Svenson (ENPC, transferred to USNM). Paratype (male): Dominican Republic: Cordillera Central: Constanza, 1300 m, 12 July 1978, Fortuné Chalumeau (CRAAG).

### Oedudes anulatus

Lingafelter sp. n.

urn:lsid:zoobank.org:act:167E8FC9-C0F9-43E0-AC83-F3BD559B316D

http://species-id.net/wiki/Oedudes_anulatus

Figs 1a ; 3b-d ; Map 1

#### Diagnosis.

This species is easily distinguished from all other Hispaniolan hemilophines by the bidentate elytral apices and bold red or orange pattern of pubescence on the pronotum and elytra. This species is similar to the Cuban *Oedudes ramsdeni* (Fisher), but differs in having the pronotal black spots at the middle and posterior portions of the pronotum (not concentrated only at the base as in *Oedudes ramsdeni*); the legs, including the femora, most of the tibiae, and most of the tarsi pale reddish (not dark reddish-brown to black as in *Oedudes ramsdeni*); the black fascia at the base of the elytron is large and extends to the suture and laterally down the length of the elytron (in *Oedudes ramsdeni*, it is relatively small and does not extend to the suture or lateral margin); and in having the antennae bicolored with pale annulations at the bases of at least antennomeres 2–6 (uniformly black in all other species of *Oedudes*).

#### Description.

Size: 9.0–12.0 mm long; 2.9–4.2 mm wide between humeri. *Head* with dense vestiture of appressed yellow, orange, or red setae, thickened at base, almost scale-like, dense throughout except on gena and posterior to upper eye lobes. Punctures indistinct or hidden by pubescence except on fronto-clypeal margin. Frons not bulging, level to slightly convex between eyes, without medial impression. Gena below lower eye lobe and mandibular base about one-half height of lower eye lobe; frontal-genal ridge absent; ante-clypeal sulcus obsolete. Eye large, slightly bulging laterally on lower lobe, finely faceted, upper lobe connected to lower lobe by 3–4 facets at narrowest point, lower lobe much larger than upper lobe, occupying about one-half of head from lateral view. Interantennal region not impressed, antennal tubercles weakly elevated. Antenna slender, extending beyond elytral apex by 1 antennomere in females (males unknown). Antenna with moderately dense, appressed, short pubescence of two colors, white and black, and scattered, sparse, long setae on venter of basal segments. Antennomeres mostly black with pale yellow or orange annulations on basal portions of at least 2–6. Antennomere 3 very long (longer than scape + 2; nearly as long as 4+5), subsequent antennomeres gradually decreasing in length. *Prothorax* cylindrical, slightly broader than long (1.8–2.5 mm long; 2.1–3.0 mm wide), distinctly narrower than elytral base, with very slight lateral protuberance at middle, densely covered with red or orange scale-like pubescence (fading to yellow in some pinned specimens) covering most of integument, sparse punctures visible in regions with black maculae around middle and base; pronotum without dorsal calli or tubercles, about one-fifth length of body. Prosternum integument dark brown, with sparse, short, white and translucent setae. Prosternal process broadly expanded at apex, closing procoxal cavities posteriorly. *Elytron* with distinct punctures strongest at base, becoming shallower at middle, mostly absent at apical third, dense, scale-like red or orange pubescence (fading to yellow in some dried specimens) present in bold pattern around scutellum and in transverse, slightly posteriorly angled fascia at middle and near apex, otherwise, elytra black. Humerus strongly projecting anterolaterally, often with extreme apex glabrous. Elytral apices bidentate, with concavity between sutural and apicolateral points. Elytron 6.8–9.0 mm long; 1.5–2.1 mm wide; elytral length/width: 4.2–4.5. *Scutellum* broadly rounded posteriorly, densely covered in orange or red (fading to yellow). *Legs* with femora and tibiae sublinear, only weakly thickened apically. Metafemora short, extending to about third ventrite. Moderately dense, translucent or pale pubescence on tibiae; femora sparsely pubescent. Legs pale orange or pale testaceous throughout except meso- and metafemur, tibial apices, and apices of tarsomeres which are dark brown to black. *Venter* mostly densely pubescent and dark brown or yellow-orange with denser patches of reddish-orange (fading to yellow in some dried specimens) pubescence on posterolateral margins of metasternum and ventrites 2–4 or 2–5. Apex of fifth ventrite of females broadly truncate with small median notch (males unknown).

#### Etymology.

The specific epithet is a Latin adjective, nominative case, masculine gender, meaning ringed, and refers to the pale basal annulations on most antennomeres.

#### Notes.

The genus *Oedudes* is now represented by 8 species in the Neotropics and this is the first record for the genus in Hispaniola. It is known from 3 specimens.

#### Material.

Holotype (female): Dominican Republic: Peravia Prov., 5 km W of road to El Rio, S. of Pedregal, 19°05.092'N, 70°35.864'W, 52 m, 23 June 2005, Steven W. Lingafelter (USNM). Paratypes (2 females): Dominican Republic, Pico Duarte Trail, 3300’, Los Tablones - day coll., 19°08.222'N, 70°27.736'W, 29 June 2004, D. Perez (USNM); Dominican Republic, La Vega Province, Parque Nacional Armando Bermudez, 1–3 km along trail W of La Cienaga, 900–1100 m, June 22, 2005, Specimen ID 7643, Nearns & Lingafelter (ENPC).

### Calocosmus contortus

Lingafelter sp. n.

urn:lsid:zoobank.org:act:CC5ABEFE-3753-496D-AA89-968A8D931E2C

http://species-id.net/wiki/Calocosmus_contortus

Fig. 4a-d ; Map 1

#### Diagnosis.

This species, like *Calocosmus thonalmus*, is very distinctive since it has a similarly contorted elytral apex. It differs in having the middle antennomeres (4–7) fulvous annulate at the basal one-fourth to one-half (the antennae are uniformly black in *Calocosmus thonalmus*); the lower eye lobe much larger, the genal region below it is less than one-third height of lower eye lobe (nearly as high as the lower eye lobe in *Calocosmus thonalmus*); in having the elytral apex with a minor pubescent ridge between the two major costal ridges (*Calocosmus thonalmus* has a simple glabrous depression between the two major costal ridges); a relatively long metasternal setal brush (this setal brush, apparently only developed in males of some species of *Calocosmus*, is much shorter in *Calocosmus thonalmus*); and in having longer and more slender femora and tibiae (the metafemur extends to the posterior margin of the third ventrite in *Calocosmus contortus* but only to the anterior margin of third ventrite in *Calocosmus thonalmus*).

#### Description.

Size: 8.2 mm long; 2.5 mm wide between humeri. *Head* with dense vestiture of very short, ashy-white setae, slightly thickened at base, but not obscuring surface, along with scattered long, dark setae on frons. Sparse, mostly non-contiguous punctures scattered throughout head. Frons not bulging, moderately concave between eyes, with division by median groove extending to vertex. Gena below lower eye lobe and mandibular base about one-third height of lower eye lobe; frontal-genal ridge very short, extending vertically for a short distance toward eye margin from genal margin. Anteclypeal sulcus absent. Eye large, bulging laterally on lower lobe beyond plane of head, finely faceted, upper lobe connected to lower lobe by 3 facets at narrowest point, lower lobe much larger than upper lobe, occupying nearly one-half of head from lateral view. Interantennal region impressed, antennal tubercles slightly elevated. Antenna moderately stout, short, surpassing elytral apex by about 2 antennomeres in males (females unknown). Antenna with vestiture of semi-appressed, dense, short, black pubescence and scattered, sparse, long, dark setae, especially at antennomere apices and mesal margins. Antennomeres black, except scape which is reddish-brown and 4–7 which have orange basal annulations. Antennomere 3 longer than scape + 2 but shorter than 4+5, subsequent antennomeres short and gradually decreasing in length or subequal. *Prothorax* cylindrical, broader than long (1.3 mm long; 1.8 mm wide), distinctly narrower than elytral base, with only slight middle swelling, densely covered with short whitish-gray setae, however inconspicuous and not obscuring integument. Pronotum with overall orange appearance, without maculae. Pronotum with distinct, large, scattered, non-contiguous punctures. Pronotum about one-sixth length of body. Prosternum inconspicuously pubescent with short, orange or translucent setae. Prosternal process very narrow between protuberant procoxae, broadly expanded at apex, closing procoxal cavities posteriorly. *Elytron* with distinct, dense punctures that terminate at anterior margin of dark, apical macula, with areas of dense, short, erect, velvety white, pubescence that does not obscure surface and sparse, longer, erect, black setae scattered on basal two-thirds. Elytron bicolored: basal one-half orange, apical one-half iridescent purplish-black. Purplish-black elytral apex highly modified and contorted with 2 elevated costae at middle with crest of erect, short, black setae. Intercostal region between them is level, with small region of erect, black setae. Humerus not projecting anterolaterally, with pubescence similar to adjacent regions. Elytral apices narrowly rounded to suture, without spines. Elytron 5.9 mm long; 1.3 mm wide; elytral length/width: 4.5. *Scutellum* narrowly subtruncate posteriorly, with short, black setae and orange ground color. *Legs* with tibiae weakly thickened apically. Femora and tibiae slender, elongate; metafemur surpassing third ventrite. Legs with white and translucent pubescence not obscuring surface, becoming most dense and darker on tibiae. Femora orange; tibiae dark reddish-brown; tarsomeres dark reddish-brown at apices. *Venter* mostly sparsely and inconspicuously pubescent (not obscuring surface). Venter orange throughout except for apex of fifth ventrite of male which is darkened. Apex of fifth ventrite of males broadly truncate with small median notch (females unknown).

**Figure 4.**
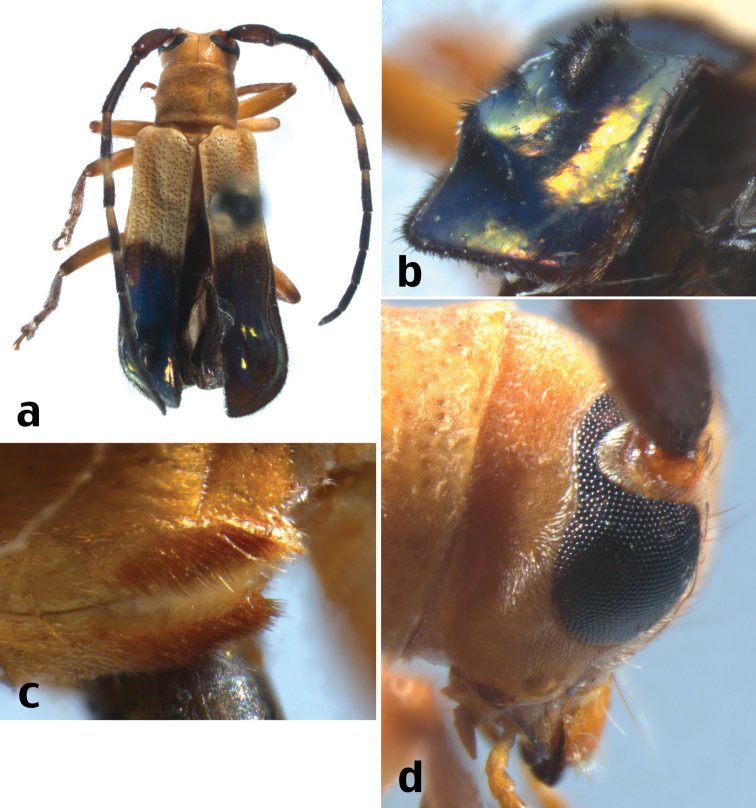
*Calocosmus contortus* Lingafelter, sp. n., holotype: **a** dorsal habitus **b** elytral apex **c** metasternal pubescent tufts **d** lateral view of head.

#### Etymology.

The species epithet is a Latin noun, nominative case, masculine gender that refers to the contorted elytral apex.

#### Notes.

This species, along with *Calocosmus thonalmus*, is among the most highly morphologically evolved members of the Batesian mimicry complex with the lycid beetle genus *Thonalmus* (Fig. 2b). It is known from one specimen.

#### Material.

Holotype (male): Dominican Republic, [San Cristóbal Province], Colonia Ramfis, 3 April 1953, J. A. Ramos (USNM).

### Calocosmus punctatus

Lingafelter sp. n.

urn:lsid:zoobank.org:act:428B2358-139A-490C-8177-A4525D6A330F

http://species-id.net/wiki/Calocosmus_punctatus

Fig. 5a ; Map 2

#### Diagnosis.

This species is distinct from other species of *Calocosmus* (and other Hispaniolan hemilophines) by the distinct, dense punctures throughout the elytra, the all black antennae with very narrowly white annulate antennomeres, and the nearly completely black elytra except for the pattern of orange maculae as described below.

#### Description.

Size: 12.3 mm long; 4.9 mm wide between humeri. *Head* with dense vestiture of very short, appressed off-white setae, thickened at base, almost scale-like but not obscuring surface, most dense on frons and around antennae, less dense elsewhere. Large, well-defined, non-contiguous punctures scattered throughout head. Frons not bulging, level between eyes with division by a median groove extending to vertex. Gena below lower eye lobe and mandibular base a little more than one-half height of lower eye lobe; frontal-genal ridge incomplete, extending briefly at 45 degree angle between eye margin and clypeal margin. Anteclypeal sulcus absent. Eye not large, not bulging laterally on lower lobe beyond plane of head, finely faceted, upper lobe connected to lower lobe by 3 facets at narrowest point, lower lobe larger than upper lobe, occupying about one-fourth of head from lateral view. Interantennal region not impressed, antennal tubercles very slightly elevated. Antenna moderately stout, short, not attaining elytral apex in female (males unknown). Antenna with vestiture of appressed, dense, short pubescence of two colors, white and black, and scattered, sparse, long setae, especially at antennomere apices and mesal margins. Antennomeres black with exception of extreme bases that are annulate with appressed, white, setae. Antennomere 3 not very long (shorter than scape + 2; nearly as long as 4+5, which are short), subsequent antennomeres very short and subequal or gradually decreasing in length. *Prothorax* cylindrical, slightly broader than long (2.7 mm long; 4.0 mm wide), distinctly narrower than elytral base, with small post-lateral protuberance, densely covered with appressed, short, yellowish-orange scale-like pubescence, however not obscuring integument. Pronotum with overall orange appearance with ovate, black divided macula at middle and slightly darker patches anterior to elytral base. Pronotum with distinct, large, non-contiguous punctures, without dorsal calli or tubercles, but with swelling posterior to middle. Pronotum a little less than one-fourth length of body. Prosternum inconspicuously pubescent with short, appressed, orange or yellow setae. Prosternal process broadly expanded at apex, closing procoxal cavities posteriorly. *Elytron* with distinct, dense punctures throughout except on extreme apex, covered in dense, short pubescence, but not obscuring surface. Elytral color black, except for orange pattern as follows: base around scutellum, along suture to middle, along part of epipleuron, and small antemedial spot. Humerus moderately projecting anterolaterally, with extreme apex glabrous. Elytral apices broadly rounded to suture, without spines. Elytron 8.9 mm long; 2.5 mm wide; elytral length/width: 3.6. *Scutellum* broadly rounded posteriorly, with dense but inconspicuous pubescence that does not obscure orange ground color. *Legs* with femora and tibiae weakly thickened apically. Metafemora short, just barely extending to third ventrite. Legs with white and translucent pubescence not obscuring surface, becoming most dense at apex of tibiae. Legs orange except for apical one-half of tibiae and all of tarsi which are black. *Venter* mostly densely but inconspicuously pubescent (not obscuring surface). Venter orange throughout except for dark spot on metasternum, part of metepisternum, and lateral margin of ventrites 1–3. Apex of fifth ventrite of females broadly truncate with very small median notch (males unknown).

**Figure 5.**
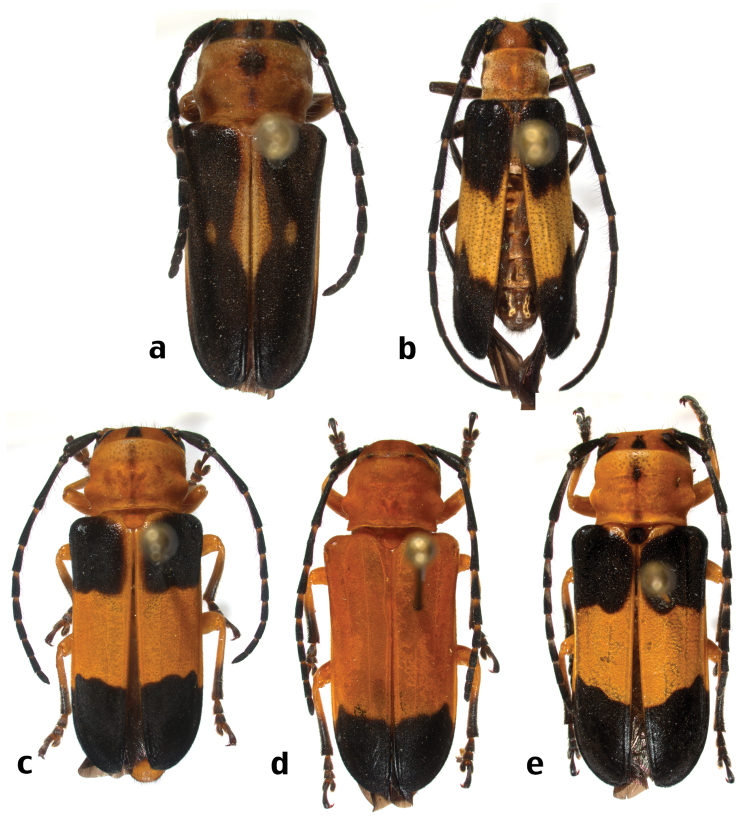
New species of Hispaniolan Hemilophini (not to scale): **a** *Calocosmus punctatus* Lingafelter, sp. n., holotype **b** *Calocosmus rawlinsi* Lingafelter, sp. n., holotype **c** *Calocosmus robustus* Lingafelter, sp. n., holotype **d** *Calocosmus robustus* Lingafelter, sp. n., paratype **e** *Calocosmus robustus* Lingafelter, sp. n., paratype.

**Map 2.**
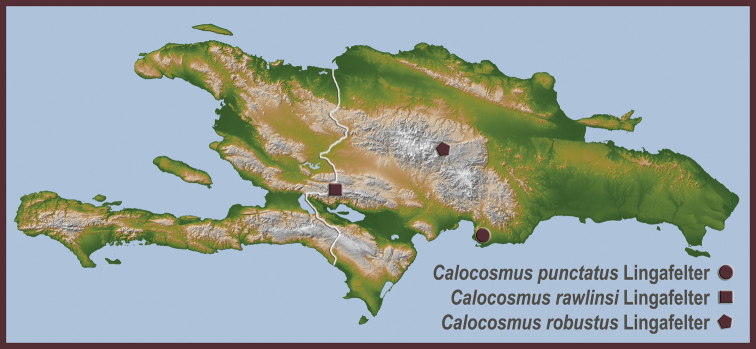
Distributional locality records for new species of Hispaniolan Hemilophini.

#### Etymology.

The specific epithet is a Latin adjective, nominative case, masculine gender that refers to the dense, conspicuous elytral punctures.

#### Notes.

This species is known from a single specimen.

#### Material.

Holotype (female): Dominican Republic: Peravia Prov., 12.4 km E. Rio Ocoa, 3 July 1992, M. A. & R. O. Ivie, collectors (WIBF, transferred to USNM).

### Calocosmus rawlinsi

Lingafelter sp. n.

urn:lsid:zoobank.org:act:CB50E9D9-2BD8-408B-B971-9A1BB583EC0F

http://species-id.net/wiki/Calocosmus_rawlinsi

Fig. 5b ; Map 2

#### Diagnosis.

This slender species is distinguished from all other Hispaniolan hemilophines by the combination of having the legs nearly completely dark reddish-brown to black, base of elytron, including scutellum, nearly completely dark, apical region of elytron dark, mostly pubescent and impunctate, and humeri not projecting. It is most similar to *Calocosmus chevrolati* Fisher, but in that species the femora and scutellum are fulvous-orange and the apical black elytral macula is mostly punctate.

#### Description.

Size: 9.6 mm long; 2.7 mm wide between humeri. *Head* with dense vestiture of very short, appressed yellow-orange setae, not thickened at base, not obscuring surface, most dense on frons and antennal tubercles, less dense elsewhere. Scattered long, black setae present on frons. Large, well-defined, non-contiguous punctures scattered throughout head. Frons not bulging, slightly concave between eyes with poorly defined median groove extending to vertex. Gena below lower eye lobe and mandibular base about one-half height of lower eye lobe; frontal-genal ridge incomplete, extending dorsally but not contacting eye margin. Anteclypeal sulcus absent. Eye large, slightly bulging laterally on lower lobe beyond plane of head, finely faceted, upper lobe connected to lower lobe by 3 facets at narrowest point, lower lobe larger than upper lobe, occupying about one-third of head from lateral view. Interantennal region weakly impressed, antennal tubercles weakly elevated. Antenna moderately slender, exceeding elytral apex by a little more than 2 antennomeres in male (females unknown). Antenna with vestiture of appressed, dense, short, black pubescence and fringe of long setae, especially at antennomere apices and mesal margins. Antennomeres black with exception of extreme bases of 3–7 that are orange-fulvous annulate. Antennomere 3 not very long (only slightly longer than scape + 2; shorter than 4+5), subsequent antennomeres subequal or gradually decreasing in length. *Prothorax* cylindrical, broader than long (1.5 mm long; 2.0 mm wide), distinctly narrower than elytral base, with small lateral protuberance at middle, densely covered with appressed, short, yellowish-orange scale-like pubescence, however not completely obscuring integument. Pronotum with overall orange appearance, without maculae. Pronotum with distinct, large, non-contiguous punctures on most of disc, without dorsal calli or tubercles. Pronotum a little less than one-sixth length of body. Prosternum inconspicuously pubescent with short, appressed, orange or yellow setae. Prosternal process between strongly protuberant procoxae, broadly expanded at apex, closing procoxal cavities posteriorly. *Elytron* with distinct, dense punctures throughout but absent from most of apical black maculate region, covered in dense, short pubescence of two colors, black and yellow-orange, but not obscuring surface. Elytral color with black and orange pattern as follows: most of basal third black, most of middle third orange, most of apical third black. Humerus not projecting anterolaterally, without glabrous region. Elytral apices narrowly rounded to suture, without spines. Elytron 7.3 mm long; 1.4 mm wide; elytral length/width: 5.2. *Scutellum* broadly rounded posteriorly, with moderately dense, black pubescence and dark ground color. *Legs* with tibiae weakly thickened apically. Metafemur extending to third ventrite. Legs mostly covered with black setae, not obscuring surface, becoming most dense at apex of tibiae. Legs black throughout except for coxa and anterior face of profemur which are reddish-brown. *Venter* mostly densely but inconspicuously pubescent (not obscuring surface). Venter mostly orange throughout, but suffused with darker areas on some thoracic and abdominal sclerites. Apex of fifth ventrite of male rounded, without median notch (females unknown).

#### Etymology.

The species epithet is a genitive patronym in honor of John Rawlins (CMNH) for leading important expeditions to the Dominican Republic and collecting the holotype.

#### Notes.

This species is known from a single specimen.

#### Material.

Holotype (male): Dominican Republic: Elias Pina Prov., Sierra de Neiba, 9.1 km WSW Hondo Valle, 18°41'38"N, 71°46'56"W, 1856 m, 30 April 2006, J. Rawlins, J. Hyland, R. Davidson, C. Young, D. Koenig, J. Fetzner, wet montane forest, pine, hand collected, Sample 31246, Carnegie Museum Specimen Number CMNH-532,987 (CMNH).

### Calocosmus robustus

Lingafelter sp. n.

urn:lsid:zoobank.org:act:B1DCD724-CEB3-4D9A-9CDF-A9091E8109A3

http://species-id.net/wiki/Calocosmus_robustus

Fig. 5c-e ; Map 2

#### Diagnosis. 

Like *Calocosmus nigritarsis*, this is a highly polymorphic species with regard to maculations of the head, pronotum, scutellum, and elytron. This robust species is most similar to *Calocosmus melanurus* and *Calocosmus nigritarsis* in its large size and proportions. It differs from *Calocosmus melanurus* in having at least part of the tibiae and tarsi darkened (entirely fulvous in *Calocosmus melanurus*). It differs from *Calocosmus nigritarsis* in having most antennomeres with basal fulvous annulations and a pronotum either entirely fulvous or with a very small anteromedial dark macula (antennae black and most antennomeres without basal annulations; pronotum usually with a large anteromedial black macula in *Calocosmus nigritarsis*).

#### Description.

Size: 13.4–15.4 mm long; 5.2–5.9 mm wide between humeri. *Head* with dense vestiture of very short, orange pubescence that does not obscure surface, maculae of similar black pubescence present on vertex and/or posterior to upper eye lobes in some specimens. Scattered long, black setae present on frons. Large, well-defined, non-contiguous punctures scattered throughout frons and posterior to upper eye lobes. Frons not bulging, either level or slightly convex between eyes, with division by median groove extending to vertex. Gena below lower eye lobe and mandibular base about one-half height of lower eye lobe; frontal-genal ridge incomplete, extending for a short distance at 45 degree angle between eye margin and clypeal margin. Anteclypeal sulcus absent. Eye small, not bulging laterally on lower lobe beyond plane of head, finely faceted, upper lobe connected to lower lobe by 2–3 facets at narrowest point, lower lobe larger than upper lobe, occupying about one-fourth of head from lateral view. Interantennal region not impressed, antennal tubercles not or very slightly elevated. Antenna moderately stout, short, not attaining elytral apex in females (males unknown). Antenna with vestiture of appressed, dense, short, translucent pubescence (also white pubescence in one specimen) and scattered, sparse, long black and translucent setae, especially at antennomere apices and mesal margins. Antennomeres black with exception of extreme bases that are orange-fulvous annulate (sometimes with appressed, white setae). Antennomere 3 short (only slightly longer than scape + 2; subequal to or slightly longer than 4+5 which are short), subsequent antennomeres subequal or gradually decreasing in length. *Prothorax* cylindrical, broader than long (2.8–3.1 mm long; 4.0–4.5 mm wide); distinctly narrower than elytral base, with pronounced lateral protuberance at middle, densely covered with short orange-red setae, however not obscuring integument. Pronotum with overall orange appearance, immaculate or with small, ill-defined black macula at center of disc. Pronotum with distinct, large, mostly non-contiguous punctures throughout, without dorsal calli or tubercles, but with swelling at middle. Pronotum about one-fifth length of body. Prosternum inconspicuously pubescent with short, orange or red setae. Prosternal process between strongly protuberant procoxae, broadly expanded at apex, closing procoxal cavities posteriorly. *Elytron* with distinct, dense punctures, becoming shallow or absent by apical third, covered in dense, separate regions of short, velvet-like orange or red pubescence, but not obscuring surface. Elytral color variable, with black and orange or red regions as follows: basal and apical one-third black with middle one-third orange or red, or basal two-thirds orange or red with apical one-third black. Humerus moderately or weakly projecting anterolaterally, partially denuded of pubescence at apex. Elytral apices broadly rounded to suture, without spines. Elytron 9.9–11.3 mm long; 2.6–3.0 mm wide; elytral length/width: 3.7–3.8. *Scutellum* broadly rounded posteriorly, with inconspicuous pubescence that does not obscure orange or black ground color. *Legs* with tibiae weakly thickened apically. Metafemur short, barely reaching third ventrite. Legs with white and translucent pubescence not obscuring surface, becoming most dense at apex of tibiae. Legs orange except for apical one-half of tibiae and all or part of tarsi which are black. *Venter* mostly densely but inconspicuously pubescent, not obscuring surface. Venter orange throughout or with dark spot on metasternum and occasionally metepisternum. Apex of fifth ventrite of females rounded, with median notch (males unknown).

#### Etymology.

The species epithet is a Latin adjective, nominative case, masculine gender that refers to the robustness of the individuals.

#### Notes.

This species is known from 3 female specimens.

#### Material.

Holotype (female): Dominican Republic, La Vega Province, Parque Nacional Armando Bermudez, km 1–3 along trail W of La Ciénaga, 900–1100 m, 19°01.753'N, 70°54.654'W, 2 July 2010, N. E. Woodley (USNM). Paratypes (2 females): Dominican Republic, La Vega Province, Parque Nacional Armando Bermudez, km 1–3 along trail W of La Ciénaga, 900–1100 m, [no coordinates], 7 June 2005, SpecID: 7062, Gino Nearns (ENPC); same data but 24 June 2005, SpecID: 7608, Nearns & Lingafelter (USNM).

### Calocosmus thonalmus

Lingafelter sp. n.

urn:lsid:zoobank.org:act:5DB1E36E-1CF4-4BF3-A046-1F85CB825917

http://species-id.net/wiki/Calocosmus_thonalmus

Fig. 6a-d ; Map 3

#### Diagnosis.

This species is very distinctive since it, along with *Calocosmus contortus*, are the only ones with a highly modified elytral apex with elevated, pubescent ridged costae and deep intercostal spaces, giving a contorted appearance. It is distinguished from *Calocosmus contortus* by its uniformly black antennae (antennomeres 4–7 fulvous at the base in *Calocosmus contortus*); smaller lower eye lobe that is far removed from the genal base (gena is about one-third of the height of the lower eye lobe in *Calocosmus contortus*); elytra with two apical pubescent costae separated by a concave depression (a third minor costa is present between the two major ones in *Calocosmus contortus*); relatively short metasternal setal brush (this setal brush, apparently only developed in males of some species of *Calocosmus*, is much longer in *Calocosmus contortus*); and in its much shorter, thickened legs with the metafemur barely reaching the third ventrite (extending to the posterior margin of the third ventrite in *Calocosmus contortus*.) It is also superficially similar to some specimens of *Calocosmus hispaniolae* since both species possess a dark, shiny apical area of the elytron (with elevated costae in some specimens of *Calocosmus hispaniolae*), but they are never convoluted with pubescent crests as in *Calocosmus thonalmus*.

#### Description.

Size: 7.0–7.9 mm long; 2.1–2.7 mm wide between humeri. *Head* with dense vestiture of very short, ashy-white setae, slightly thickened at base, but not obscuring surface, along with scattered long, translucent or dark setae on frons. Numerous well-defined, mostly non-contiguous punctures scattered throughout head. Frons not bulging, either level or slightly concave between eyes, with division by median groove extending to vertex. Gena below lower eye lobe and mandibular base about two-thirds height of lower eye lobe; frontal-genal ridge very short, extending for a short distance vertically toward eye margin from genal margin. Anteclypeal sulcus absent. Eye small to moderate sized, very weakly bulging laterally on lower lobe beyond plane of head, finely faceted, upper lobe connected to lower lobe by 2–3 facets at narrowest point, lower lobe larger than upper lobe, occupying about one-fourth of head from lateral view (slightly smaller in females). Interantennal region not impressed, antennal tubercles not elevated. Antenna moderately stout, short, surpassing elytral apex by less than 1 antennomere in females and a little more than 2 antennomeres in males. Antenna with vestiture of semi-appressed, dense, short, black pubescence and scattered, sparse, long setae, especially at antennomere apices and mesal margins. Antennomeres black, without annulations. Antennomere 3 longer than scape + 2 but shorter than 4+5, subsequent antennomeres short and gradually decreasing in length. *Prothorax* cylindrical, broader than long (1.3–1.6 mm long; 1.6–2.0 mm wide), distinctly narrower than elytral base, with middle swelling and lateral protuberance, densely covered with short yellowish-orange setae, however not obscuring integument. Pronotum with overall orange appearance, without maculae. Pronotum with distinct, large, mostly non-contiguous punctures throughout. Pronotum about one-fifth length of body. Prosternum inconspicuously pubescent with short, orange or translucent setae. Prosternal process broadly expanded at apex, closing procoxal cavities posteriorly. *Elytron* with distinct, dense punctures that terminate at anterior margin of dark, apical macula, with areas of dense, short, erect, velvety white, pubescence that does not obscure surface and sparse, longer, erect, black setae scattered on basal two-thirds. Elytron bicolored: slightly more than basal one-half orange, slightly less than apical one-half iridescent purplish-black. Purplish-black elytral apex highly modified and contorted with 2 elevated costae at middle with crest of erect, short, black setae. Intercostal regions concave and mostly glabrous. Humerus not projecting anterolaterally, partially denuded of pubescence at apex. Elytral apices narrowly rounded to suture, without spines. Elytron 4.8–5.7 mm long; 1.0–1.4 mm wide; elytral length/width: 4.0-4.8. *Scutellum* broadly subtruncate posteriorly, mostly glabrous with orange ground color. *Legs* with tibiae weakly thickened apically. Femora and tibiae short; metafemur barely reaching third ventrite. Legs with white and translucent pubescence not obscuring surface, becoming most dense at apex of tibiae. Femora orange; tibiae orange to black; tarsi dark orange to black. *Venter* mostly sparsely and inconspicuously pubescent, not obscuring surface. Venter orange throughout except for apex of fifth ventrite of both sexes which is piceous. Apex of fifth ventrite of females broadly truncate with very small median notch, broadly rounded with relatively larger notch in males.

**Figure 6.**
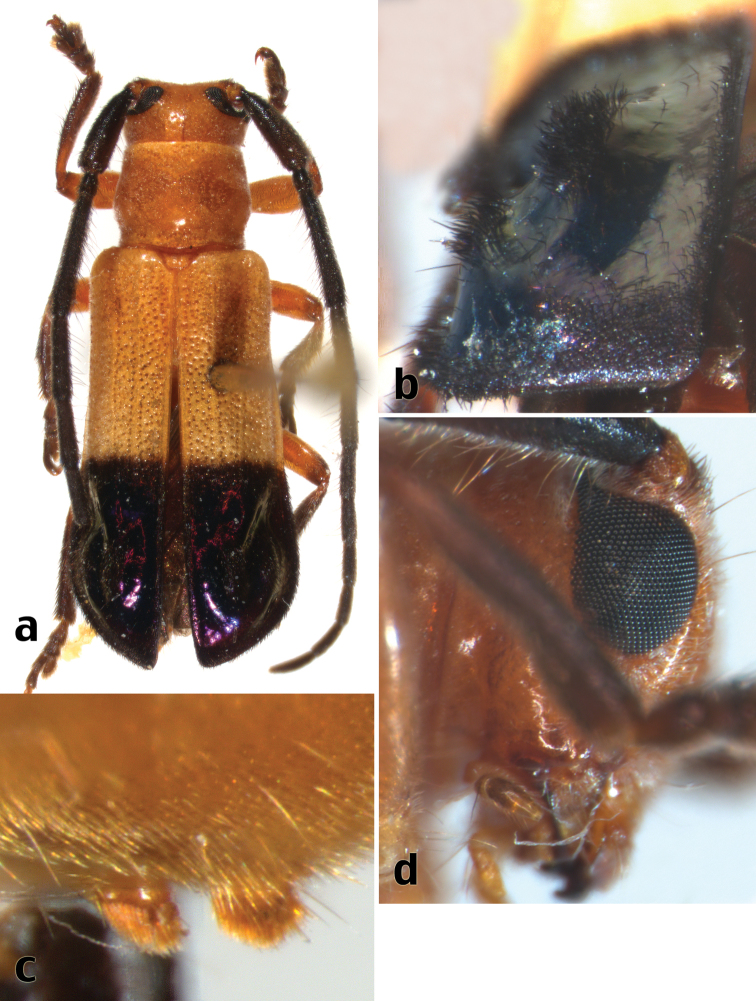
*Calocosmus thonalmus* Lingafelter, sp. n., holotype: **a** dorsal habitus **b** elytral apex **c** metasternal pubescent tufts **d** lateral view of head.

**Map 3.**
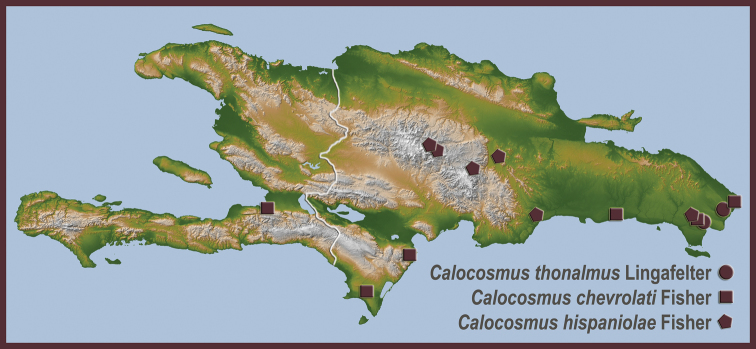
Distributional locality records for new species and new distributional records for previously described Hispaniolan Hemilophini.

#### Etymology.

The species epithet is a Latin noun, nominative case, masculine gender that refers to the similarity of this species to members of the lycid genus, *Thonalmus* Bourgeois.

#### Notes.

This species, along with *Calocosmus contortus*, is a highly modified member of the Batesian mimicry complex with the lycid beetle genus *Thonalmus* (Fig. 2b). It is known from two specimens.

#### Material.

Holotype (male): Dominican Republic, La Altagracia Province, El Veron, road to Hoyo Azul, 25–40 m, beating, SpecID: 6701, Nearns & Lingafelter, 26 June 2005 (ENPC, transferred to USNM). Paratype (1 female): Dominican Republic, La Altagracia Province, Parque Nacional del Este, 2.9 km SW Boca de Yuma, 18°21'51"N, 68°37'05"W, 11 m, 28 May 2004, C. Young, J. Rawlins, J. Fetzner, C. Nunez, semihumid dry forest, limestone, UV light, Sample 52114, CMNH 396,805 (CMNH).

### Calocosmus chevrolati

Fisher

http://species-id.net/wiki/Calocosmus_chevrolati

Fig. 7a-b ; Map 3

#### Diagnosis.

This species is variable with regard to the presence or absence of dark maculae at the elytral base and or humerus. It is similar to *Calocosmus hispaniolae* in size and proportions, but the apical black elytral macula is matte and lacks the metallic purple iridescence that is present in *Calocosmus hispaniolae*. This region is also very pubescent and punctate while in *Calocosmus hispaniolae*, large areas are devoid of pubescence and punctation. It is somewhat similar to *Calocosmus rawlinsi*, but differs in having punctures in the apical dark portion of the elytron and in having the femora fulvous (dark reddish-brown to black in *Calocosmus rawlinsi*).

**Figure 7.**
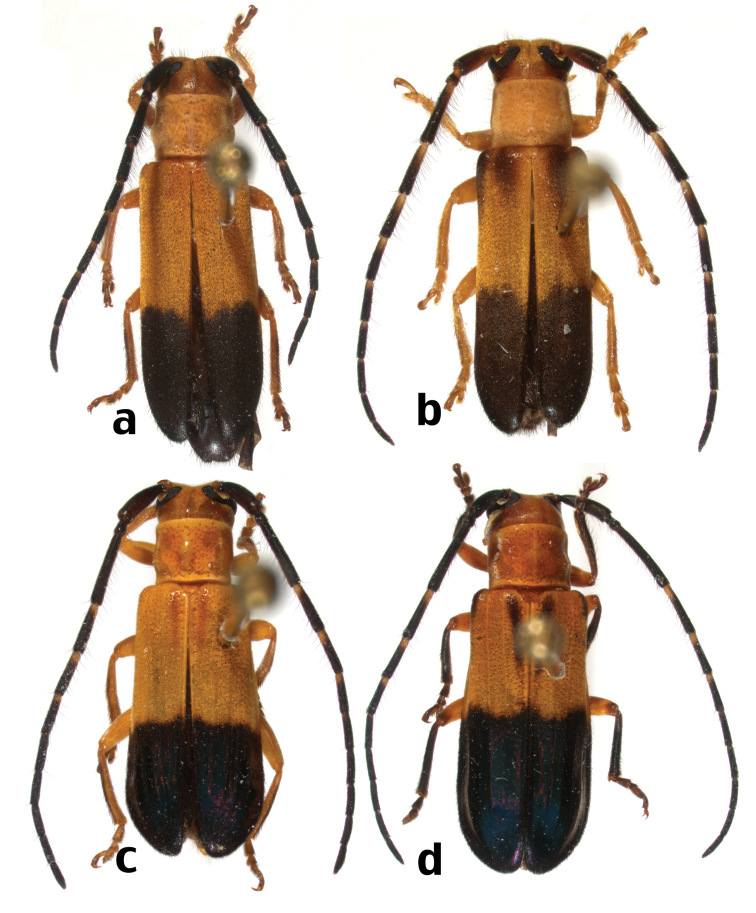
Dorsal habitus of *Calocosmus* species: **a** *Calocosmus chevrolati* Fisher (morphotype 1) **b** *Calocosmus chevrolati* Fisher (morphotype 2) **c** *Calocosmus hispaniolae* Fisher (morphotype 1) **d** *Calocosmus hispaniolae* Fisher (morphotype 2).

#### Notes.

This species, previously known only from Cuba, is here recorded for Hispaniola, a new island record. Haiti and the Dominican Republic represent new country records.

#### New distributional data.

Haiti, Port au Prince, 1899, R. D. Crew (1 specimen, USNM); Dominican Republic, La Altagracia Province, Parque Nacional del Este, Boca de Yuma, 18°21.508'N, 68°36.956'W, 19 July 2004, N. Woodley, S. Lingafelter & 20 July 2004, D. Perez, S. Lingafelter (2 specimens, USNM); Dominican Republic, La Altagracia Prov, Punta Cana, near Ecological Reserve, 0-5 meters, 18°30.477'N, 68°22.499'W, 14 June 2005, S. Lingafelter & 2 July 2006, S. Lingafelter (2 specimens, USNM); Dominican Republic, Barahona Province, 11 km S. Barahona, May 6–17, 1985, E. Giesbert, coll. (FSCA); Dominican Republic, Pedernales, PN Jaragua, 99 m, UV light, 3 km S Los Tres Charcos, Spec. ID 7034, Nearns and Lingafelter, 16 June 2005 (1 specimen, ENPC); Dominican Republic, San Pedro de Macorís Province, 12 km W. San Pedro de Macorís, 5–19 May, 1985, E. Giesbert, coll. (FSCA).

### Calocosmus hispaniolae

Fisher

http://species-id.net/wiki/Calocosmus_hispaniolae

Fig. 7c-d ; Map 3

#### Diagnosis.

In size and proportions, this species is similar to *Calocosmus chevrolati*. It is easily distinguished by having the dark apex of the elytron with a shiny, metallic purple iridescence (black with a matte finish in *Calocosmus chevrolati*). Also, this apical region has areas lacking pubescence and punctures (densely punctate and pubescent in *Calocosmus chevrolati*). The metallic elytral apex sometimes has elevated costae making specimens similar to *Calocosmus thonalmus* and *Calocosmus contortus*, however, the elytral apex is never contorted to that extreme and lacks the costal pubescent ridges characteristic of those species.

#### Notes.

This species is known only from Hispaniola.

#### New distributional data.

Dominican Republic, La Altagracía Province, Parque Nacional del Este, Boca de Yuma, 18°21.508'N, 68°36.956'W, 20 July 2004, N. Woodley, S. Lingafelter (1 specimen, USNM); Dominican Republic, La Vega Province, Parque Nacional Armando Bermudez, km 1–3 along trail west of La Ciénega, 1100 m, 22 June 2005, A. Konstantinov (1 specimen, USNM); Dominican Republic, Santiago Province, Parque Nacional Armando Bermudez, Rio Bao, 1212 m, 10 July 1992, M A. & R. O. Ivie (1 specimen, WIBF); Dominican Republic, La Altagracia Province, Parque del Este, 2.9 km southwest of Boca de Yuma, 18°21.51'N, 68°37.05'W, 11 m, 28 May 2004, C. Young, J. Rawlins, J. Fetzner, C. Nunez, semihumid dry forest, limestone, hand collected, 52114, CMNH 327,105 (1 specimen, CMNH); Dominican Republic, La Vega Province, 4.1 km southwest El Convento, 18°50.37'N, 70°42.48'W, 1730 m, 31 May 2003, J. Rawlins, R. Davidson, C. Young, C. Nuñez, P. Acevedo, dense secondary evergreen forest with pine, hand collected, sample 22242, CMNH 319,721 (1 specimen, CMNH); Dominican Republic, San Cristóbal Province, 10 miles north San Cristóbal, 27 August, 1967, J. C. Schaffner (1 specimen, TAMU); Dominican Republic, La Vega Province, 8 miles west Jayaco, 3 August, 1967, J. C. Schaffner (1 specimen, TAMU).

### Calocosmus janus

Bates

http://species-id.net/wiki/Calocosmus_janus

Fig. 8a ; Map 4

Calocosmus holosericeus Gahan, 1889: 395, syn. n.

#### Diagnosis.

This species is easily distinguished from all other Hispaniolan Hemilophini by the uniformly purplish-black elytra, black antennae without annulations, and pronounced elytral humeri. The only other species with mostly dark elytra are *Calocosmus punctatus*, *Calocosmus semimarginatus*, and *Calocosmus nigripennis*. *Calocosmus punctatus* is distinguished easily by having the antennae with basal annulations of gray pubescence on most antennomeres, the elytra with scattered fulvous regions, and having the elytral punctures far more dense and well defined than in *Calocosmus janus*. *Calocosmus semimarginatus* and *Calocosmus nigripennis* are easily distinguished by lacking humeral projections and by their small size (most specimens less than 8 mm long while most specimens of *Calocosmus janus* are greater than 10 mm long).

**Figure 8.**
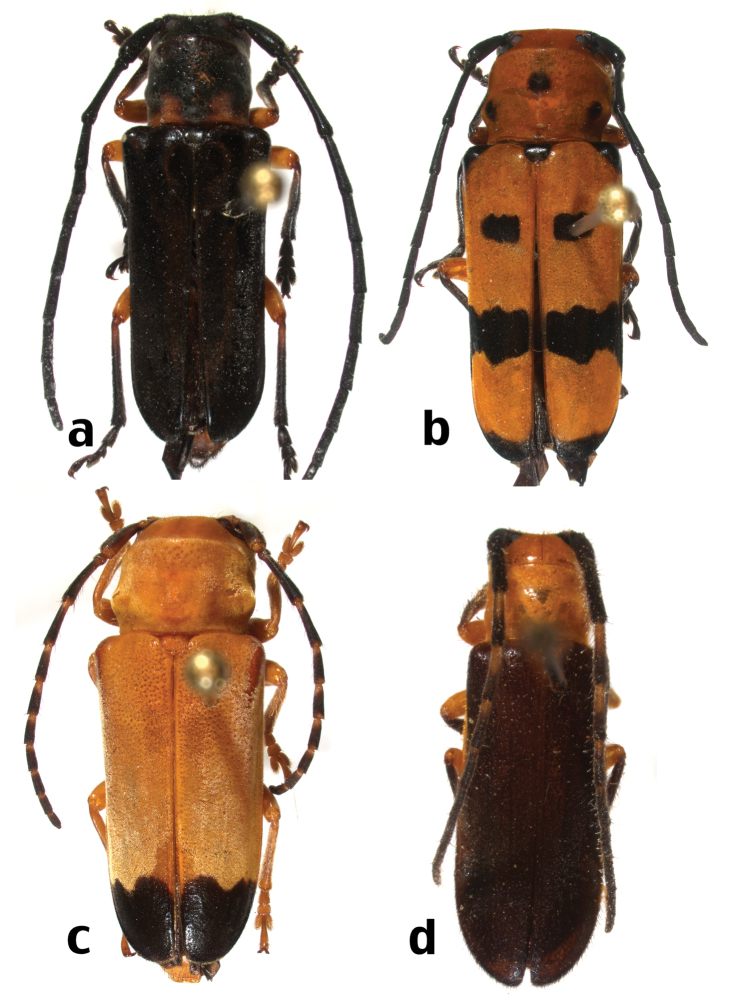
Dorsal habitus of *Calocosmus* species (not to scale): **a** *Calocosmus janus* Bates **b** *Calocosmus magnificus* Fisher **c** *Calocosmus melanurus* Gahan **d** *Calocosmus nigripennis* Chevrolat.

**Map 4.**
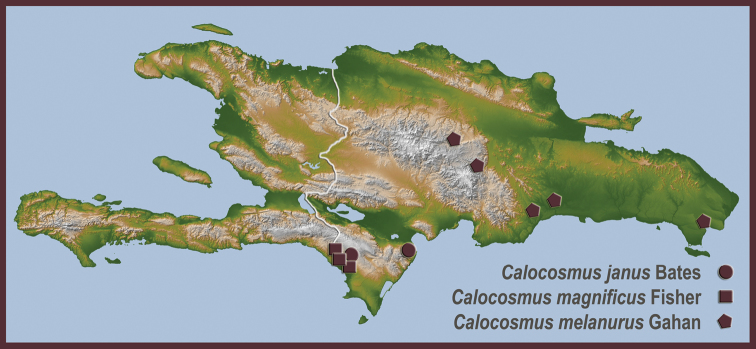
New distributional locality records for previously described Hispaniolan Hemilophini.

#### Notes.

*Calocosmus holosericeus* Gahan is a new synonym of *Calocosmus janus* Bates. Photos of the holotypes show they are nearly identical, as are the original Latin descriptions for each. *Calocosmus holosericeus* was described from Hispaniola and *Calocosmus janus* was described from Cuba. The Dominican Republic and Hispaniola therefore represent new country and island records for *Calocosmus janus*.

#### New distributional data.

Dominican Republic, Pedernales Province, Parque Nacional Sierra de Baoruco, Las Abejas, 18°09.011'N, 71°37.342'W, 1150 m, 18 June 2005, S. Lingafelter (1 specimen, USNM); Dominican Republic, Barahona Province, 4.5 km S. Barahona, 23 May 1992, R. Turnbow (1 specimen, RHTC).

### Calocosmus magnificus

Fisher

http://species-id.net/wiki/Calocosmus_magnificus

Figs 1b , 8b ; Map 4

#### Diagnosis.

This large species is easily recognized by its striking, bold red or orange coloration with black maculae on the elytra and pronotum. It is the only species with the apical black macula of the elytron restricted to less than the apical one-eighth, a small, humeral macula, and the pronotum dorsally with three small, dark maculae (one at center, and one at each side of center). The postmedial black macula can range in size from a spot less than half the width of the elytron to a transverse band extending the full width of the elytron.

#### Notes.

This is perhaps the most strikingly colored *Calocosmus* known. In life, specimens have a vivid red color with bold, black maculae. Unfortunately, this fades to pale orange or yellow in most pinned specimens. This species was described from Haiti, and recorded herein from the Dominican Republic for the first time.

#### New distributional data.

Dominican Republic, Pedernales Province, 13 May 2010, 1 km N. of Banano, Rio Molito, 18°09.256'N, 71°45.384'W, 290 m, Kelvin Guerrero (1 specimen, USNM); Dominican Republic, Pedernales Province, 25 km N. Cabo Rojo, 18°06.769'N, 71°37.245'W, 679 m, Kelvin Guerrero (1 specimen, USNM); Dominican Republic: Pedernales, Sierra de Baoruco, Aceitillar, 23.6 km NE Pedernales, 18°09'23"N, 71°34'09"W, 1560 m., 14 June 2003, C. Young, J. Rawlins, C. Nunez, R. Davidson, P. Acevedo, M. de la Cruz, open pine forest with grassland, hand collected sample 42142, Carnegie Museum Specimen Number 318,968 (1 specimen, CMNH).

### Calocosmus melanurus

Gahan

http://species-id.net/wiki/Calocosmus_melanurus

Fig. 8c ; Map 4

#### Diagnosis.

This species is very similar to some specimens of *Calocosmus nigritarsis* and *Calocosmus robustus* with regard to dorsal coloration, size, and proportions. It is distinguished from them by having fulvous tibiae and mostly fulvous tarsi (tibial apex and tarsi, at least, dark reddish-brown to black in *Calocosmus nigritarsis* and *Calocosmus robustus*); more distinctly fulvous annulate antennomeres (at least 4–7), and a dense vestiture of ashy pubescence on the pronotum and elytra of fresh specimens (less conspicuous or absent in other species).

#### Notes.

This species has some external sexual dimorphism. Females are broader, have more pronounced lateral and dorsal pronotal swelling, and have less pronounced elytral humeri than males.

#### New distributional data.

Dominican Republic, Santo Domingo Province, Puerto Vaca, Sierra Prieta, 18°38'N, 69°57'W, 27 May 2004, R. H. Bastardo (1 specimen, USNM); Dominican Republic, La Altagracía Province, Parque Nacional del Este, Boca de Yuma, 18°21.508'N, 68°36.956'W, 3–20 m, 27 June 2005, M. L. Chamorro (1 specimen, USNM); Dominican Republic, San Cristóbal Province, Las Desanparados, San Cristóbal, August, 1987 (1 specimen, USNM); Dominican Republic, La Vega, 5.1 km. N. Manabao, 5 June 1994, R. Turnbow (1 specimen, RHTC); Dominican Republic, La Vega Province, Constanza, 17 July 1996, R. Turnbow (1 specimen, RHTC); Dominican Republic, La Vega, 2 km. N Jarabacoa, 25 May 1992, R. Turnbow (1 specimen, RHTC).

### Calocosmus nigripennis

Chevrolat

http://species-id.net/wiki/Calocosmus_nigripennis

Fig. 8d ; Map 5

#### Diagnosis.

This species, like *Calocosmus janus*, *Calocosmus punctatus*, and *Calocosmus semimarginatus*, typically has nearly completely black elytra. It is immediately distinguished from *Calocosmus janus* and *Calocosmus punctatus* by its slender form, without projecting humeri, and small size, less than 8 mm long (more than 10 mm long and much broader in *Calocosmus janus* and *Calocosmus punctatus*). Chevrolat (1862), in his original description, indicates that *Calocosmus nigripennis* can be recognized by having the pronotum transversely bisulcate, although this feature is less pronounced in the Haiti specimen than in the holotype.

**Map 5.**
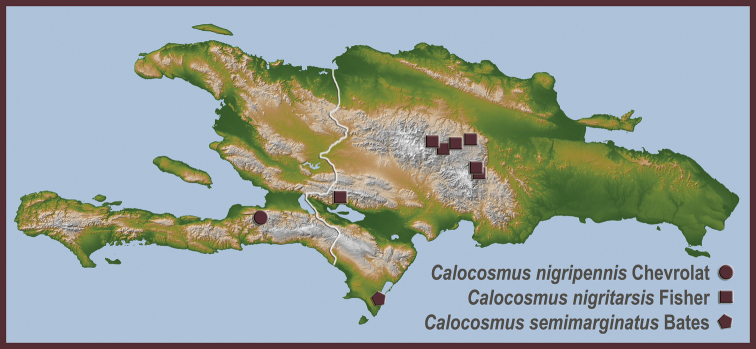
New distributional locality records for previously described Hispaniolan Hemilophini.

#### Notes.

This species, previously known only from Cuba, is now recorded from Haiti (new country and island record). Other specimens examined from Cuba, while similar in most other features, have the elytral base varying from all black to pale orange. Additional material is needed to fully understand the level of variation of this species.

#### New distributional data.

Haiti, Ouest Department, Kenscoff, 1200 m, May 25, 1984, M. Thomas (1 specimen, FSCA).

### Calocosmus nigritarsis

Fisher

http://species-id.net/wiki/Calocosmus_nigritarsis

**Figure 9.**
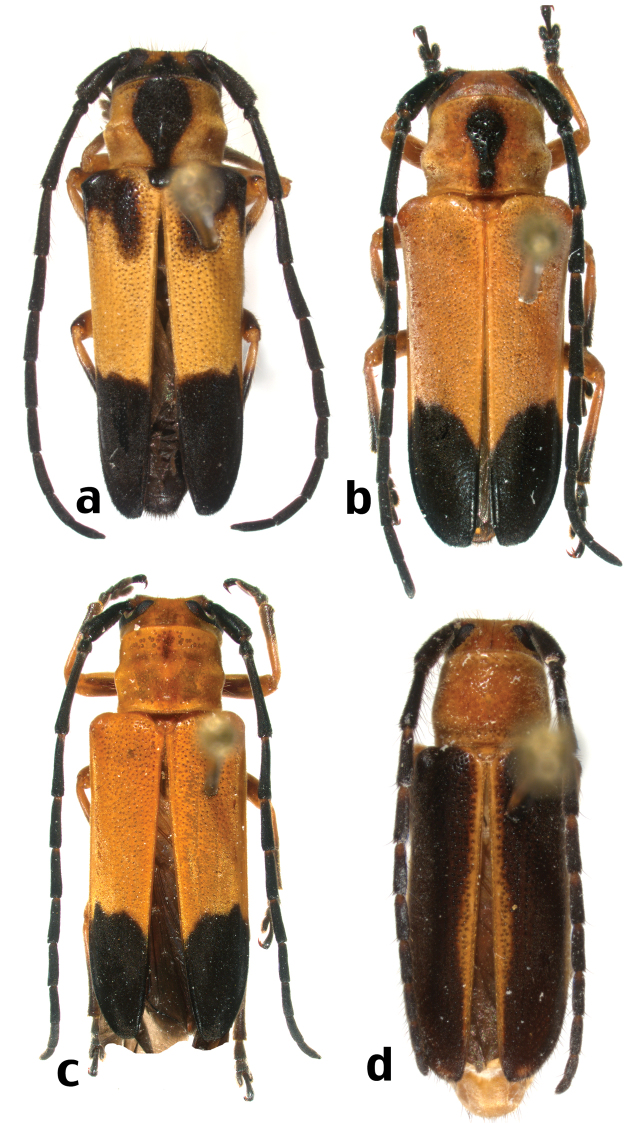
Dorsal habitus of Hispaniolan Hemilophini species (not to scale): **a** *Calocosmus nigritarsis* Fisher (morphotype 1) **b** *Calocosmus nigritarsis* Fisher (morphotype 2) **c** *Calocosmus nigritarsis* Fisher (morphotype 3) **d** *Calocosmus semimarginatus* Bates.

Figs 1c-d , 9a-c ; Map 5

#### Diagnosis.

Like *Calocosmus robustus*, this is a highly polymorphic species with regard to maculations of the head, pronotum, scutellum, and elytron. It is allied with the group of large, robust species that include *Calocosmus robustus* and *Calocosmus melanurus*. It is distinguished from *Calocosmus melanurus* by having the tibiae partially and tarsi completely piceous (the tibiae are completely and the tarsi are at least partially fulvous in *Calocosmus melanurus*). The antennae of *Calocosmus nigritarsis* are nearly completely black with, at most, only the extreme bases of a few antennomeres fulvous (*Calocosmus melanurus* and *Calocosmus robustus* have the antennal fulvous annulations more pronounced). In almost all specimens of *Calocosmus nigritarsis*, the pronotum has a large, anteromedial black macula (at most a small spot in *Calocosmus robustus* and absent in *Calocosmus melanurus*.

#### Notes.

This species was previously known from only a few specimens. Fieldwork in the Dominican Republic has produced many additional specimens that have better elucidated the level of polymorphism of the elytral and pronotal maculations.

#### New distributional data.

Dominican Republic, La Vega Province, Parque Nacional Armando Bermudez, km 1–3 along trail west of La Ciénega, 900-1100 m, Specimen ID 7609, 24 June 2005, Nearns & Lingafelter (1 specimen, USNM); Dominican Republic, Independencia Prov., Road 47, between Los Pinos and Ángel Félix, 760 meters, 18°36.986'N, 71°46.556'W, 20 June 2005, N. Woodley (1 specimen, USNM); same data, but Nearns & Lingafelter (1 specimen, ENPC); Dominican Republic, La Vega Province, Jarabacoa, La Joya, 537 m, Rancho Baiguate, 10 June 2005, G. Nearns (1 specimen, ENPC); Dominican Republic, La Vega Province, 4.1 km southwest El Convento, 18°50.37'N, 70°42.48'W, 1730 m, 31 May 2003, J. Rawlins, R. Davidson, C. Young, C. Nuñez, P. Acevedo, dense secondary evergreen forest with pine, hand collected, sample 22242, CMNH 329,960 (1 specimen, CMNH); Dominican Republic, Santiago Province, Parque Nacional Armando Bermudez, La Guacara, 19°07'N, 71°02'W, 1124 m, 10 July 1992, M. A. & R. O. Ivie (1 specimen, WIBF); Dominican Republic, La Vega Province, 5.1 km N. Manabao, 5 June 1994, R. Turnbow (1 specimen, RHTC); Dominican Republic, La Vega Province, Constanza, 17 July 199, R. Turnbow (1 specimen, RHTC).

### Calocosmus semimarginatus

Bates

http://species-id.net/wiki/Calocosmus_semimarginatus

Fig. 9d

#### Diagnosis.

This small, slender species is most similar to *Calocosmus fulvicollis* Fisher, a Cuban species, in that it has nearly uniformly dark elytra with a fulvous suture. Fisher (1925) suggested that *Calocosmus fulvicollis* could be a synonym of *Calocosmus semimarginatus*, however, the head is dark in *Calocosmus fulvicollis* but light in *Calocosmus semimarginatus* and the antennae are all black in *Calocosmus fulvicollis* but at least antennomeres 4-5 are fulvous annulate at the base in *Calocosmus semimarginatus*. Among the Hispaniolan species, *Calocosmus semimarginatus* is most similar to *Calocosmus nigripennis*, but is easily distinguished by having the elytral suture pale colored and the pronotum not transversely bisulcate.

#### Notes.

The first male specimen of this species was found in the FSCA collection. This species, formerly known only from Cuba, is here recorded for the Dominican Republic and Hispaniola (new country and island records).

#### New distributional data.

Dominican Republic, Pedernales Province, S. end of Lago de Oviedo, 26 May 1986, R. B. Miller, and L. Stange (1 specimen, FSCA).

### Paleohemilophus dominicanus

Martins & Galileo

http://species-id.net/wiki/Paleohemilophus_dominicanus

#### Diagnosis.

Although similar in size and proportions to the larger *Calocosmus* species, such as *Calocosmus robustus* and *Calocosmus nigritarsis*, it is easily distinguished from all other species and genera of Hispaniolan Hemilophini by having antennomeres 3 and 4 swollen (Martins and Galileo 1999).

#### Notes.

Known only as a fossil in Dominican amber that is at least 14 million years old, this species is extinct (Martins and Galileo 1999).

## Supplementary Material

XML Treatment for Adesmus fortunei

XML Treatment for Oedudes anulatus

XML Treatment for Calocosmus contortus

XML Treatment for Calocosmus punctatus

XML Treatment for Calocosmus rawlinsi

XML Treatment for Calocosmus robustus

XML Treatment for Calocosmus thonalmus

XML Treatment for Calocosmus chevrolati

XML Treatment for Calocosmus hispaniolae

XML Treatment for Calocosmus janus

XML Treatment for Calocosmus magnificus

XML Treatment for Calocosmus melanurus

XML Treatment for Calocosmus nigripennis

XML Treatment for Calocosmus nigritarsis

XML Treatment for Calocosmus semimarginatus

XML Treatment for Paleohemilophus dominicanus
